# A 3D-printed, personalized, biomechanics-specific beta-tricalcium phosphate bioceramic rod system: personalized treatment strategy for patients with femoral shaft non-union based on finite element analysis

**DOI:** 10.1186/s12891-020-03465-1

**Published:** 2020-07-01

**Authors:** Jian Lu, Qi-Yang Wang, Jia-Gen Sheng, Shang-Chun Guo, Shi-Cong Tao

**Affiliations:** 1 Department of Orthopaedic Surgery, Shanghai Jiao Tong University Affiliated Sixth People’s Hospital, 600 Yishan Road, Shanghai, 200233 China; 2 Department of Orthopedic Surgery, Shanghai Fengxian Central Hospital, Branch of The Sixth People’s Hospital Affiliated to Shanghai Jiao Tong University, Shanghai, 201400 China; 3 Department of Medicine, Soochou University, Suzhou, 215123 Jiangsu China; 4 Institute of Microsurgery on Extremities, Shanghai Jiao Tong University Affiliated Sixth People’s Hospital, 600 Yishan Road, Shanghai, 200233 China

## Abstract

**Background:**

Although double-plate fixation (DP), i.e., fixation with a combination of a main lateral plate (LP) and a support medial plate (MP), is a relatively mature method for treating femoral shaft non-union with bone defect causes complications. The purpose of this study was to evaluate LP fixation with a 3D-printed, personalized, biomechanics-specific β-TCP bioceramic rod system (LP + 3DpbsBRS) as an alternative with less collateral damage.

**Methods:**

Structure-specific finite element modelling was used to simulate femoral shaft non-union with bone defects and treatment with an LP only as the blank control. Then, the peak von Mises stress (VMS), the VMS distribution, and the plate displacement were determined to compare the effectiveness of LP + CBG (cancellous bone grafting), DP + CBG, and LP + 3DpbsBRS under 850 N of axial force.

**Results:**

Our results indicated that the peak VMS was 260.2 MPa (LP + 3DpbsBRS), 249.6 MPa (MP in DP + CBG), 249.3 MPa (LP in DP + CBG), and 502.4 MPa (LP + CBG). The bending angle of the plate was 1.2° versus 1.0° versus 1.1° versus 2.3° (LP + 3DpbsBRS versus MP in DP + CBG versus LP in DP + CBG versus LP + CBG).

**Conclusion:**

The 3DpbsBRS in the LP + 3DpbsBRS group could replace the MP in the DP + CBG group by providing similar medial mechanical support. Furthermore, avoiding the use of an MP provides better protection of the soft tissue and vasculature.

## Background

Fractures are orthopaedic conditions that can occur at any age and are mostly caused by high-energy trauma; approximately 1.1 to 2.9 million fractures occur per year worldwide [1]. The probability of non-union after fracture is as high as 5–10% [2, 3], and non-union occurs in 1–20% of femoral shaft fractures [4]. Treating femoral non-union causes an economic [5] and psychological burden [6] on patients and is a major challenge for orthopaedic surgeons worldwide. According to imaging features, non-union can be divided into hypertrophic non-union and atrophic non-union [7, 8]. Hypertrophic non-union, also known as mechanical non-union, is characterized by excessive bone formation and poor mechanical fixation [9]. To promote mechanical stability, the most common clinical treatments include supplemental fixation, e.g., nail dynamization [10], exchanging nailing with augmentation plating [11], and augmentation plating [12]; however, the healing rate is variable (range, 53–96%) for these procedures. Atrophic non-union is characterized by the absence of a callus and cartilage due to a lack of cells and blood supply. Therefore, the fracture site may be sclerotic or osteopenic, which may lead to mechanical instability. Herein, we focus on two issues associated with the treatment of long bone non-union: mechanical stability and biological activity [13].

Autologous bone grafts with mechanical stability are the “gold standard” for the treatment of femoral shaft non-union with bone defects due to their complete histocompatibility and strong osteoconductive, osteoinductive, and osteogenic activities [13]. The rate of complications of autologous bone grafting is as high as 23% [14]; complications include pain at the donor site, haematoma, infection, loss of sensation, scar formation, and limited source of bone [15, 16]. Donor site injury problems and complications have spurred research to identify other treatment methods. Due to its excellent rigidity and stability, fixation with double-locking compression plates is one method for addressing the instability in long bone non-union [17]. However, at the same time, the medial aspect of the femur often loses a large amount of soft tissue, which leads to a reduction in the blood supply and secondary damage to bone continuity [17, 18].

The purpose of the surgical treatment of patients with femoral shaft non-union with bone defects is to provide a rigid, stable structure and create a healthy, biological environment conducive to fracture healing [9], which often is challenging for orthopaedists. In recent years, many clinical studies [19–22] have demonstrated the efficacy of beta-tricalcium phosphate (β-TCP) bioceramics as a bone graft substitute for repairing bone defects. β-TCP bioceramics have good biocompatibility and biodegradability. Furthermore, they have excellent microporosity, which is beneficial for inducing vascularization, improving osteoconductivity, and promoting cell proliferation and differentiation. However, porous bioceramics have very weak tension, which limits their application in the treatment of bone defects in weight-bearing areas. Dense bioceramics have improved mechanical properties and the ability to degrade in vitro, which could be complementary to the low mechanical properties and high bioactivity of porous bioceramics. Thus, we designed a model in which the distribution of dense and porous bioceramics would be determined according to the stress distribution of implants used to treat bone defects. In the early stages after implantation, the dense bioceramic could provide excellent mechanical support at the site of non-union, while the porous bioceramic could induce vascularization, allowing the transport of nutrients and bone ingrowth. In the late stage after implantation, the material would gradually degrade with new bone formation, and the desired biomechanical support, at the site of the bone defect, would be provided by the reconstructed bone.

In this study, we established a standardized model of femoral shaft non-union, and then, according to the predicted stress distribution of the implants that were to be implanted into the defective bone, developed a 3D-printed, personalized, biomechanics-specific β-TCP bioceramic rod system (3DpbsBRS). In a series of follow-up finite element analyses, we evaluated the biomechanical properties of the 3DpbsBRS and determined whether fixation with the 3DpbsBRS and only a lateral plate (LP) could offer similar medial support as fixation with a double plate (DP), which involves a medial plate (MP) to provide medial support and is associated with more soft tissue injury and loss of blood supply to the periosteum. This study will provide a new perspective for follow-up studies; the 3DpbsBRS is expected to provide a personalized clinical solution for individual patients in various situations, based on the idea that the combination of predictive biomechanical computation and 3D printing technology could provide personalized mechanical support, reduced plate use, and better protection of tissue and blood vessels.

## Methods

### Ethical review

This study was approved by the Ethics Committee of the Shanghai Jiao Tong University Affiliated Sixth People’s Hospital and involved the examination of an adult volunteer with a written informed consent before the study began (sex: male, age: 20, height: 178 cm, weight: 75 kg) by enhanced computed tomography (CT) to obtain raw imaging data of a normal femur.

### Establishment of a finite element model for femoral fixation

**Fig. 1:**
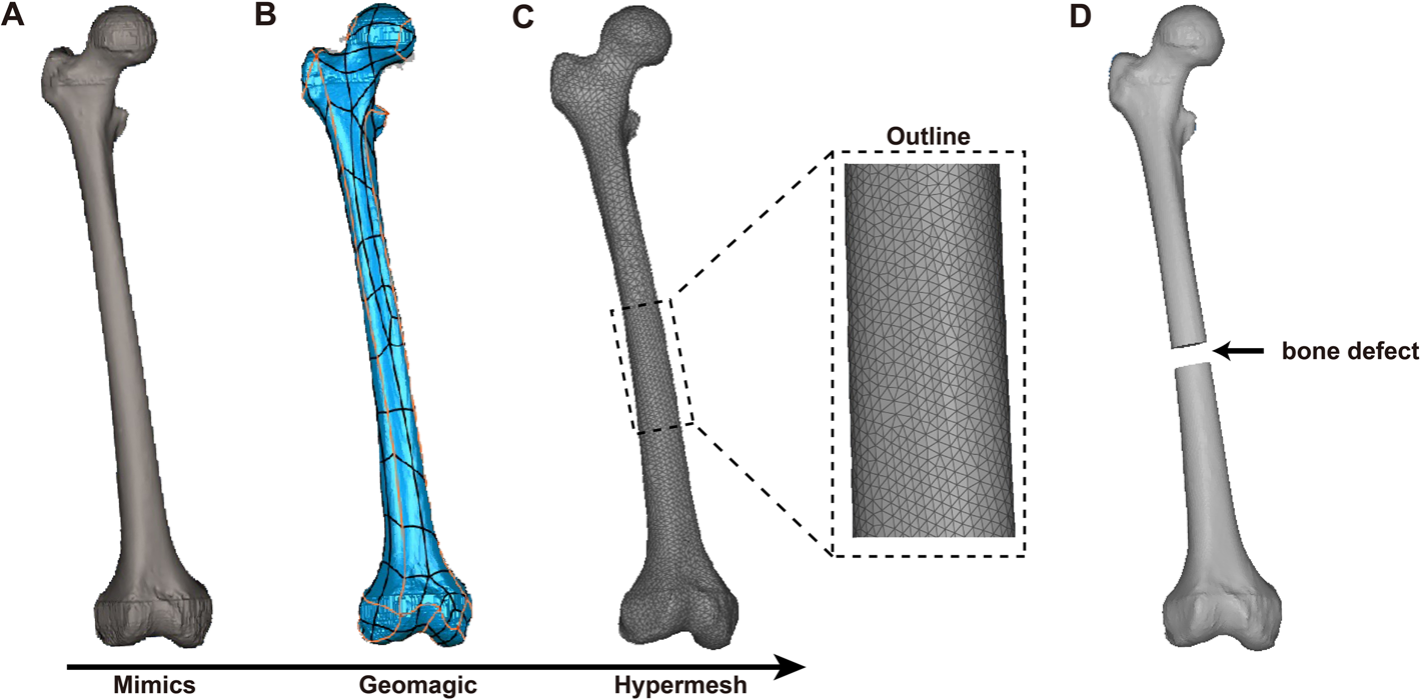
Femoral model development. **a** 3D geometric models established in Mimics. **b** Sampling and surface building for geometry in Geomagic. **c** Compiling and meshing the fundamental 3D models in HyperMesh. (D) A 15-mm transverse osteotomy plane was made at the mid-end of the femur (168 mm from the lateral femoral condyle) to simulate femoral shaft non-union with bone defects

The raw data of slices at a 0.6-mm interval in Digital Imaging and Communications in Medicine (DICOM) format were imported into Mimics 20.0 (The Materialise Group, Leuven, Belgium) to establish 3D geometric models. Herein, sampling and surface building for geometry were performed using Geomagic software. Next, the fundamental 3D models obtained were compiled and meshed in HyperMesh 14.0 (USA). Figure 1a-c depicts the sequence of software used in this study. Then, in 3-Matic 11.0 (Materialise, Leuven, Belgium), a 15-mm transverse osteotomy plane was made at the mid-end of the femur (168 mm from the lateral femoral condyle) to simulate femoral shaft non-union with bone defects (Fig. 1d).

**Fig. 2:**
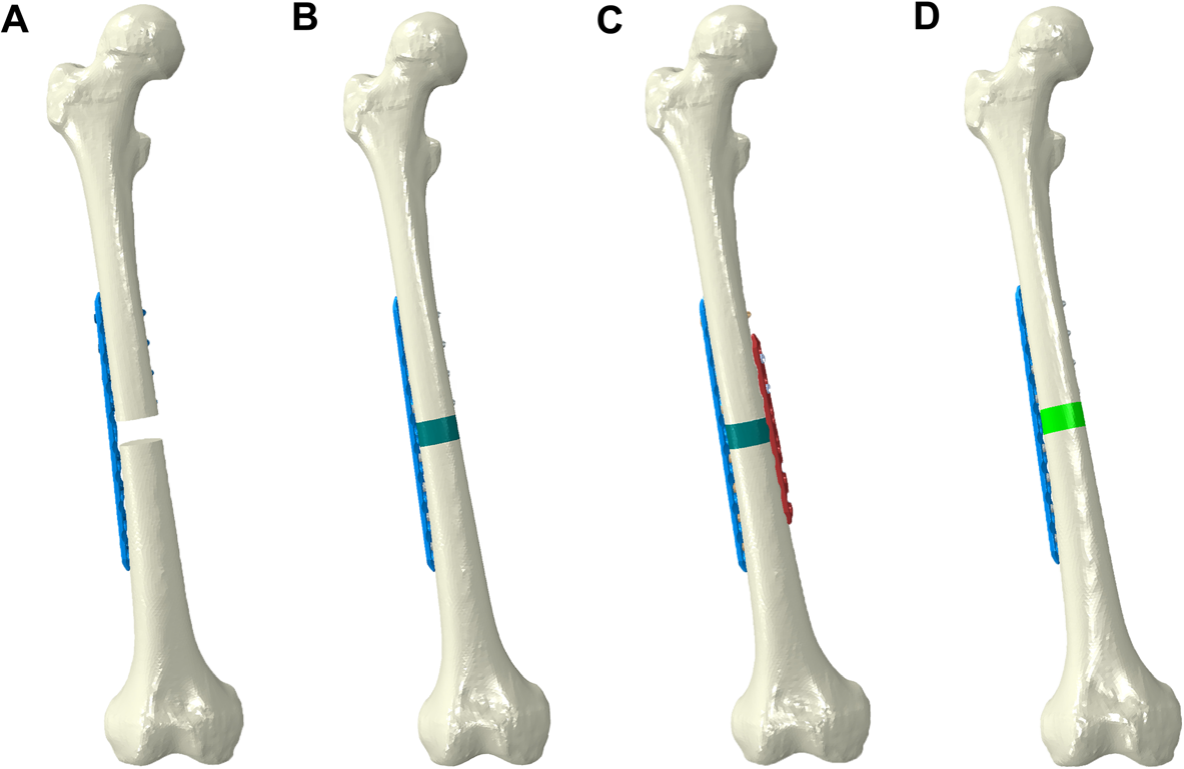
Establishment of four models (cases) for subsequent finite element analysis. **a** LP only group. **b** LP + CBG group. **c** DP + CBG group. **d** LP + 3DpbsBRS group. 3DpbsBRS, 3D-printed, personalized, biomechanics-specific β-TCP bioceramic rod system; LP, lateral plate; CBG, cancellous bone grafting; DP, double-plate

According to the blueprint provided by the manufacturer, we reconstructed the geometric 3D model of the LP, MP and screws (Synthes, 3.5-mm LCP) using Solid Works 14.0 (Solid Works Corp, Dassault Systèmes, Concord, MA, USA). The plates and the femur were assembled into four case models in 3-Matic 11.0 (Fig. 2). The threaded surface of the screws was replaced by a smooth surface; the size of the surface corresponded to the average diameter of the screw provided by the manufacturer [4].

Case 1 (LP only group: lateral main plate only): a complete femoral defect of 15 mm, with fixation of the lateral femur with a 9-hole, 3.5-mm LCP (Fig. 2a).

Case 2 (LP + CBG group: lateral main plate with cancellous bone grafting): same as case 1 with the addition of filling the defect with cancellous bone (Fig. 2b).

Case 3 (DP + CBG group: double plates with cancellous bone grafting): same as case 2 with the addition of a 6-hole, 3.5-mm LCP to the medial femoral aspect (Fig. 2c).

**Fig. 3:**
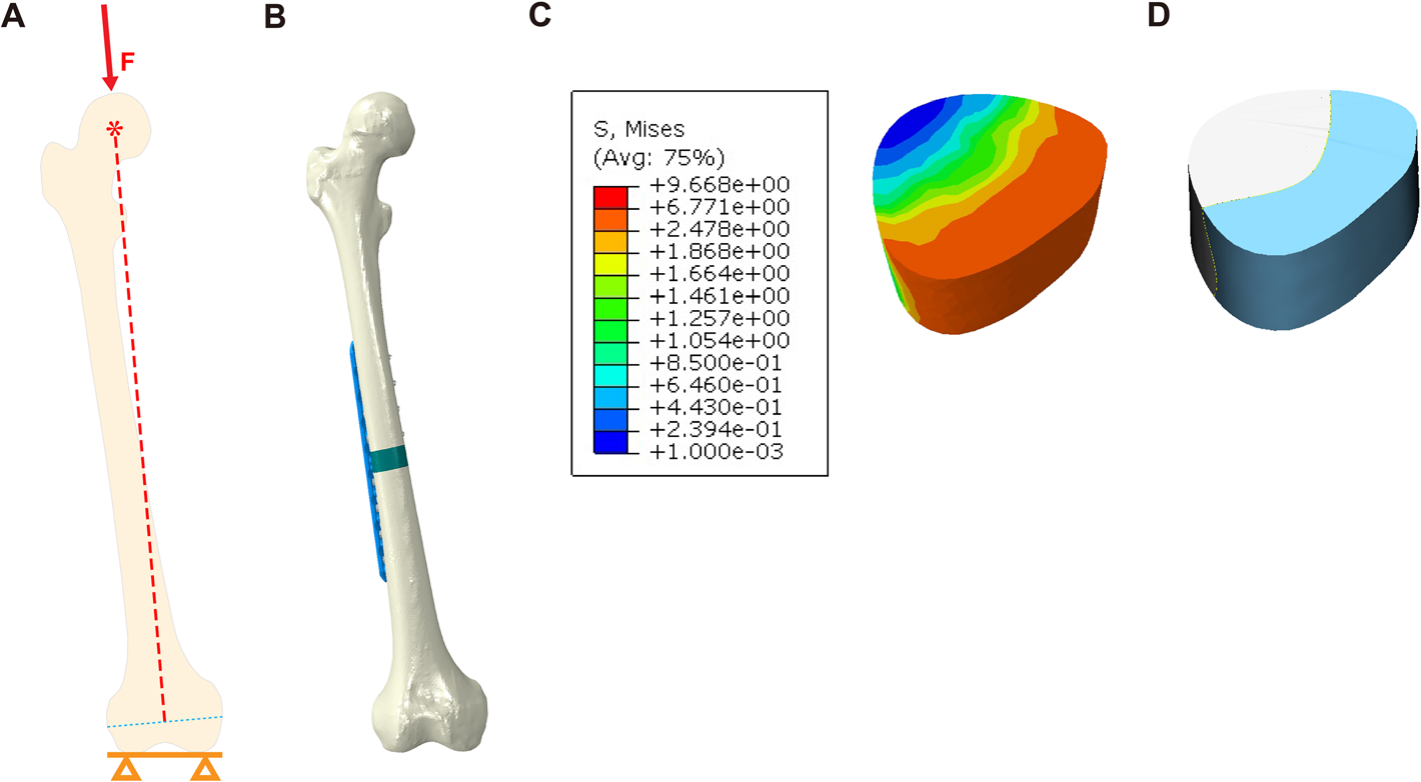
Establishment of the 3DpbsBRS. **a** Schematic of the loading force from the focal point of the femoral head to the midpoint of the femoral condyle. **b** The case 2 model was used for the finite element analysis. **c** von Mises stress distribution of cancellous bone. **d** Customized 3DpbsBRS according to the stress distribution of cancellous bone. 3DpbsBRS, 3D-printed, personalized, biomechanics-specific β-TCP bioceramic rod system

Case 4 (LP + 3DpbsBRS group: lateral main plate with 3D-printed, personalized, biomechanics-specific β-TCP bioceramic rod system): fixation with a 3.5-mm LCP on the lateral femur and filling of the defect with the 3DpbsBRS, unlike in case 2 (Fig. 2d). The steps for 3DpbsBRS acquisition were as follows: select the case 2 model of LP + CBG at the non-union bone ends for finite element analysis (Fig. 3b) and obtain the stress distribution of the grafted cancellous bone (Fig. 3c). According to the stress distribution, design the BRS with porous bioceramic when the stress is less than 2 MPa and dense bioceramic when the stress is greater than 2 MPa, yielding the 3DpbsBRS (Fig. 3d).

All of these case models underwent follow-up biomechanical simulation. Subsequently, the combined fixation and femoral model were meshed at 1 mm using HyperMesh 14.0 (USA). Finally, the combined models were imported into Abaqus 6.14 (Dassault Systèmes, USA) to generate a finite element model for mechanical analysis.

### Material properties and value assignment for finite element analysis

Based on earlier findings, the femur and strut were assumed to be linear, isotropic and elastic [23]. The LCP and screws were made of a titanium alloy (Ti-6AL-4 V). Values for the β-TCP BRS were based on data provided by Shanghai Bio-Lu Biomaterials Co., Ltd. (Shanghai, China). The porous bioceramic had a porosity of approximately 70%, a pore size of approximately 500 μm, and a pore interconnect diameter of approximately 150 μm. Table 1 shows the elastic modulus and Poisson ratio of the material, 3DpbsBRS model, and bone.

**Table 1:** Material attributes for value assignment in the finite element models (Ti-6AL-4 V, cortical, trabecular, porous ceramic granules and dense ceramic granules)

Components	Ti-6AL-4 V	Bone		β-TCP Bioceramic	
		Cortical	Trabecular	Porous ceramic granules	Dense ceramic granules
E (GPa)	105	16.7	0.155	0.2	7.49
Poisson ratio	0.35	0.26	0.3	0.3	0.3
Porosity				70%	5–10%
Compressive strength (MPa)				2.15	62

*β-TCP* beta-tricalcium phosphate; E, Young’s modulus

### Finite element analysis

According to previously published studies [24], it was assumed that there was frictional interaction between different parts of the model. The internal fixation was considered to be in the locked state, so the interface of the screw and the LP was set to be bonded, screws were tied to the bone, therefore not allowing any movement between those parts. The coefficient of friction between the cortical bone and the cancellous graft, bone and bone graft were both 0.46, and the coefficient of friction between bone and the steel plate was 0.3 [25]. To prevent rigid body motions during analysis, and all nodes on the distal surface of the femur were placed under a 0 degree-of-freedom constraint [26], under boundary conditions. Then, 850 N was applied to the centre of the femoral head of the finite element model, which was equivalent to 100% of the body weight (Fig. 3a) [27, 28]. According to Eberle et al. [14], the force vector pointed laterally 13° on the coronal plane and 8° on the sagittal plane.

### Main outcome measures

Three parameters were used to capture mechanical factors involved in fixation stability and fracture healing: the peak von Mises stress (VMS) of the implant, the VMS distribution of the implants and the displacement and deformation of the model.

## Results

The counts of element and node of four models were shownin the Table 2. The peak VMS of the plate was concentrated on the surface of the plate near the bone defect. The four fixation models showed great differences in the stress distribution.

**Table 2:** The counts of element and node of four models

Model	LP-only	LP + CBG	DP + CBG	LP + 3DpbsBRS
Element	366,127	371,747	393,283	371,747
Node	84,237	85,605	92,020	85,605

*3DpbsBRS* 3D-printed, personalized, biomechanics-specific β-TCP bioceramic rod system; *LP* lateral plate; *CBG* cancellous bone grafting; *DP* double-plate

**Fig. 4:**
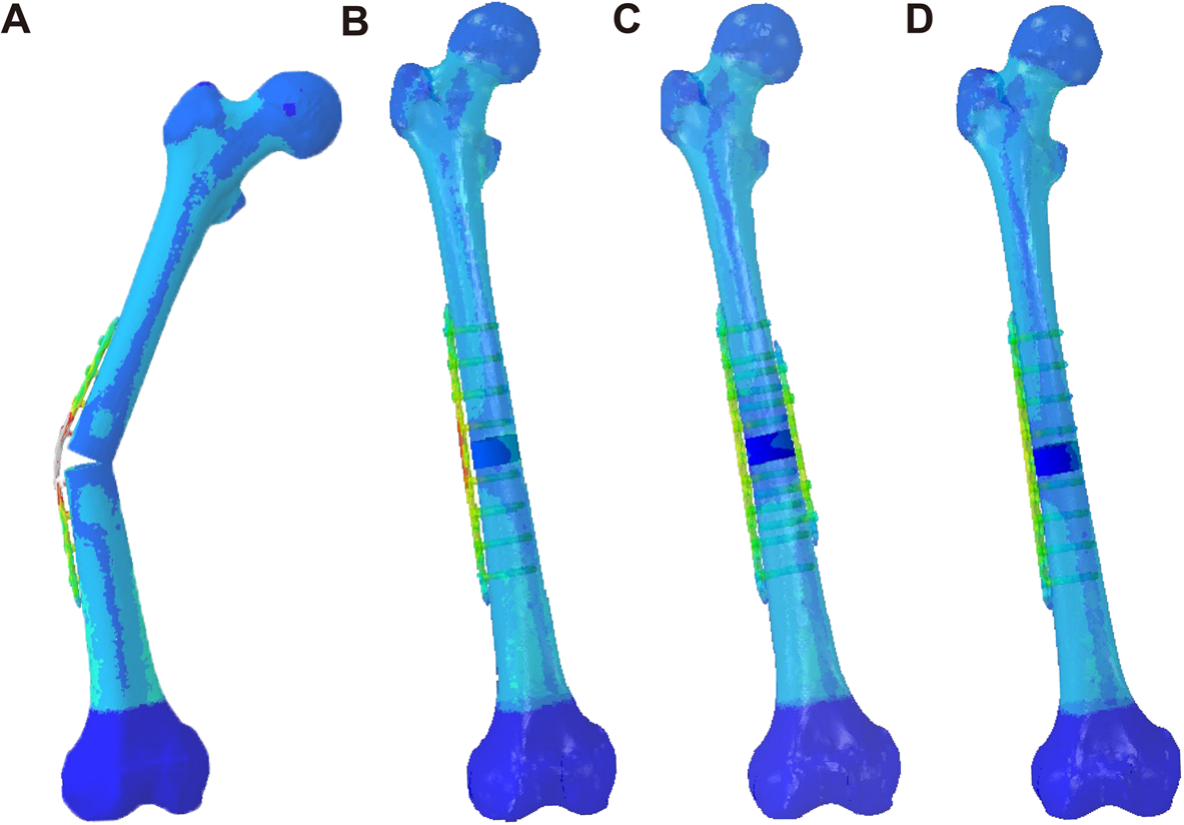
General observation of the stress distribution and deformation. **a** LP only group. **b** LP + CBG group. **c** DP + CBG group. **d** LP + 3DpbsBRS group. 3DpbsBRS, 3D-printed, personalized, biomechanics-specific β-TCP bioceramic rod system; LP, lateral plate; CBG, cancellous bone grafting; DP, double-plate

**Fig. 5:**
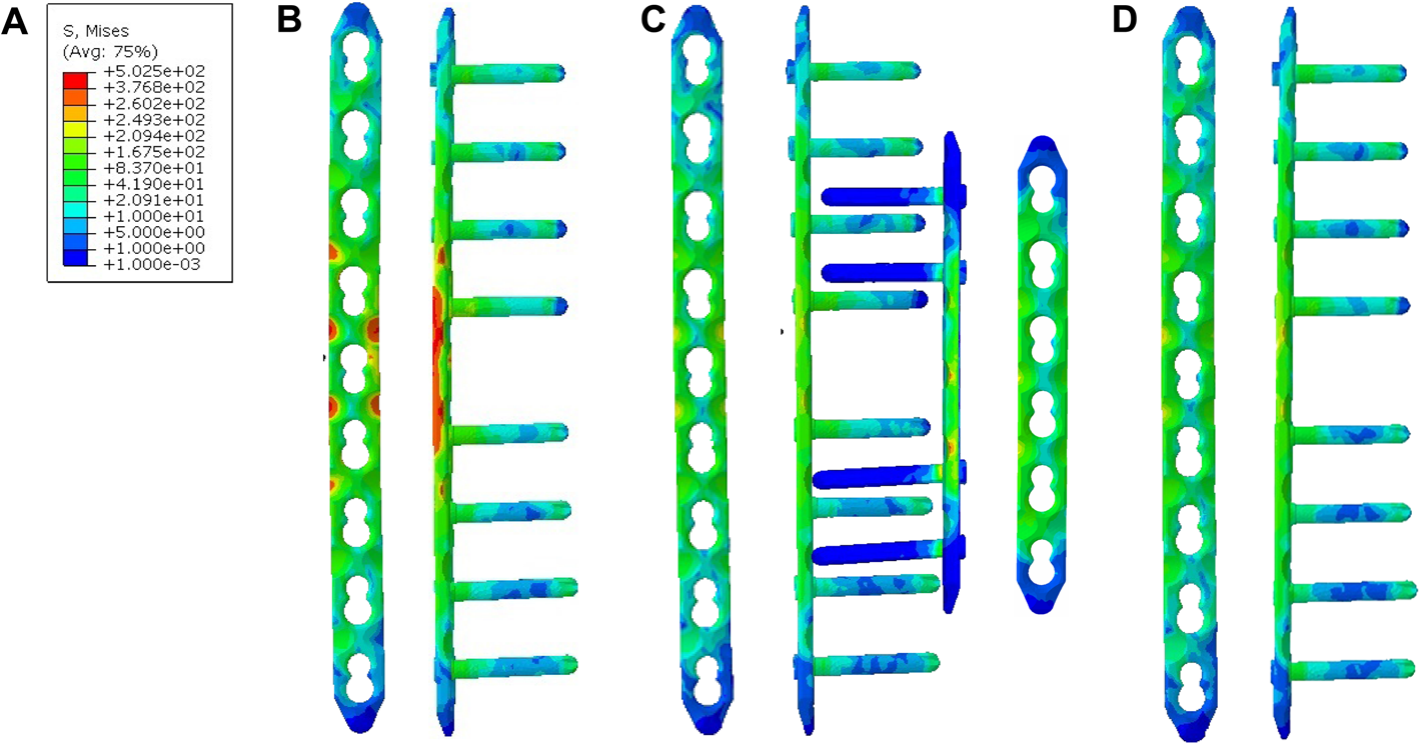
VMS distribution in the plate. **a** Unified scale for the VMS distribution. **b** LP + CBG group. **c** DP + CBG group. **d** LP + 3DpbsBRS group. VMS, von Mises stress; 3DpbsBRS, 3D-printed, personalized, biomechanics-specific β-TCP bioceramic rod system; LP, lateral plate; CBG, cancellous bone grafting; DP, double-plate

Specifically, during computational simulation, the LP-only group simulated a bone defect after fracture that was prone to fixation failure (Fig. 4a). In this model of a bone defect treated without grafting that showed failure under 850 N of axial force, specific values could not be calculated. The stress in the LP + CBG group was approximately 2 times higher than that in the DP + CBG and LP + 3DpbsBRS groups. The peak VMS of the LP in the LP + CBG group was 502.4 MPa, compared with 249.3 MPa and 260.2 MPa in the DP + CBG and LP + 3DpbsBRS groups, respectively (Figs. 5 and 7a). In the DP + CBG group, some of the stress was dissipated by the MP, which showed a peak VMS of 249.6 MPa.

**Fig. 6:**
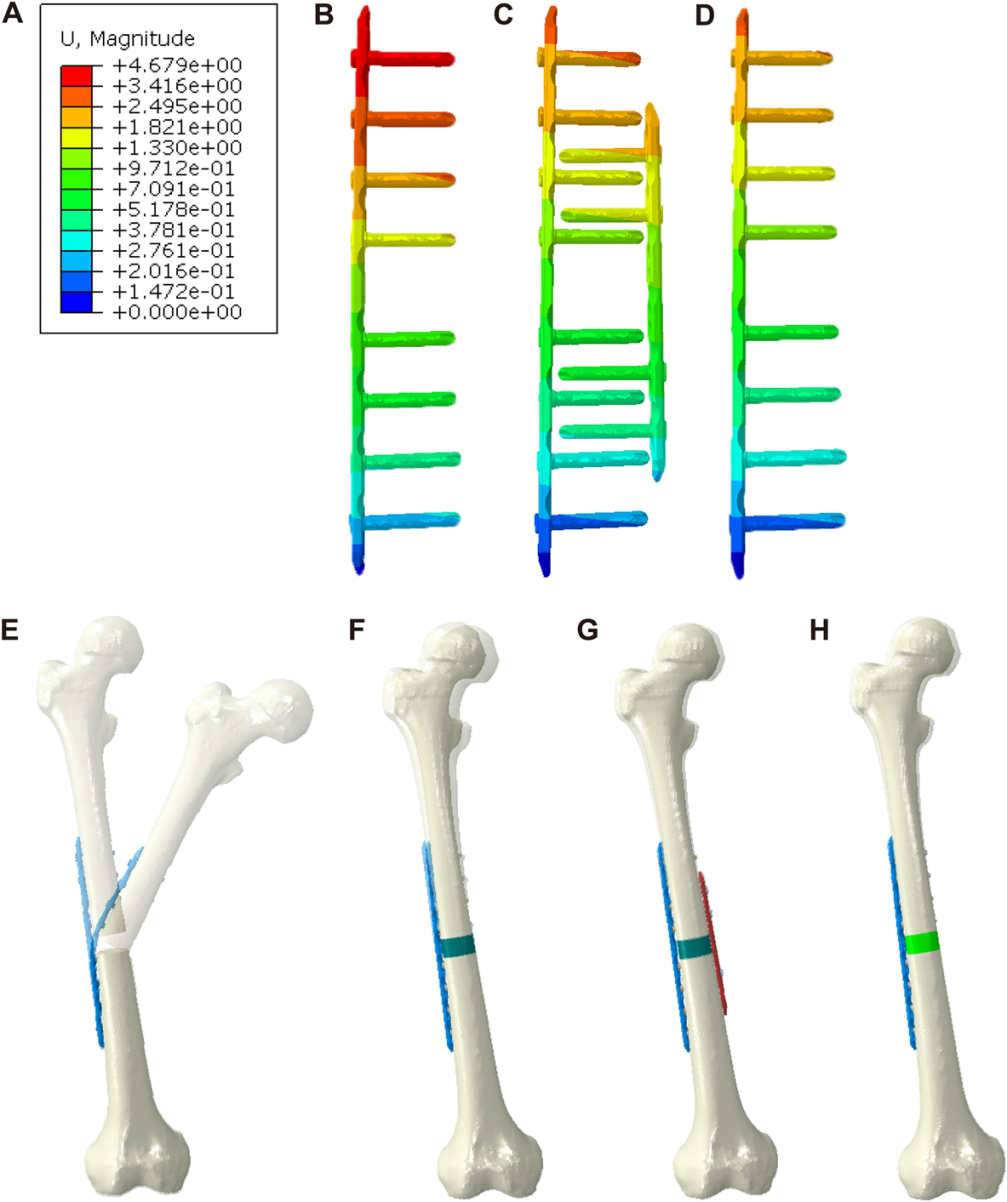
Deformation conditions in the four models (cases). **a** Unified scale for plate deformation. **b** Plate deformation in the LP + CBG group. **c** Plate deformation in the DP + CBG group. **d** Plate deformation in the LP + 3DpbsBRS group. **e** Visualized general model of displacement in the LP only group. **f** Visualized general model of displacement in the LP + CBG group. **g** Visualized general model of displacement in the DP + CBG group. **h** Visualized general model of displacement in the LP + 3DpbsBRS group. VMS, von Mises stress; 3DpbsBRS, 3D-printed, personalized, biomechanics-specific β-TCP bioceramic rod system; LP, lateral plate; CBG, cancellous bone grafting; DP, double-plate

**Fig. 7:**
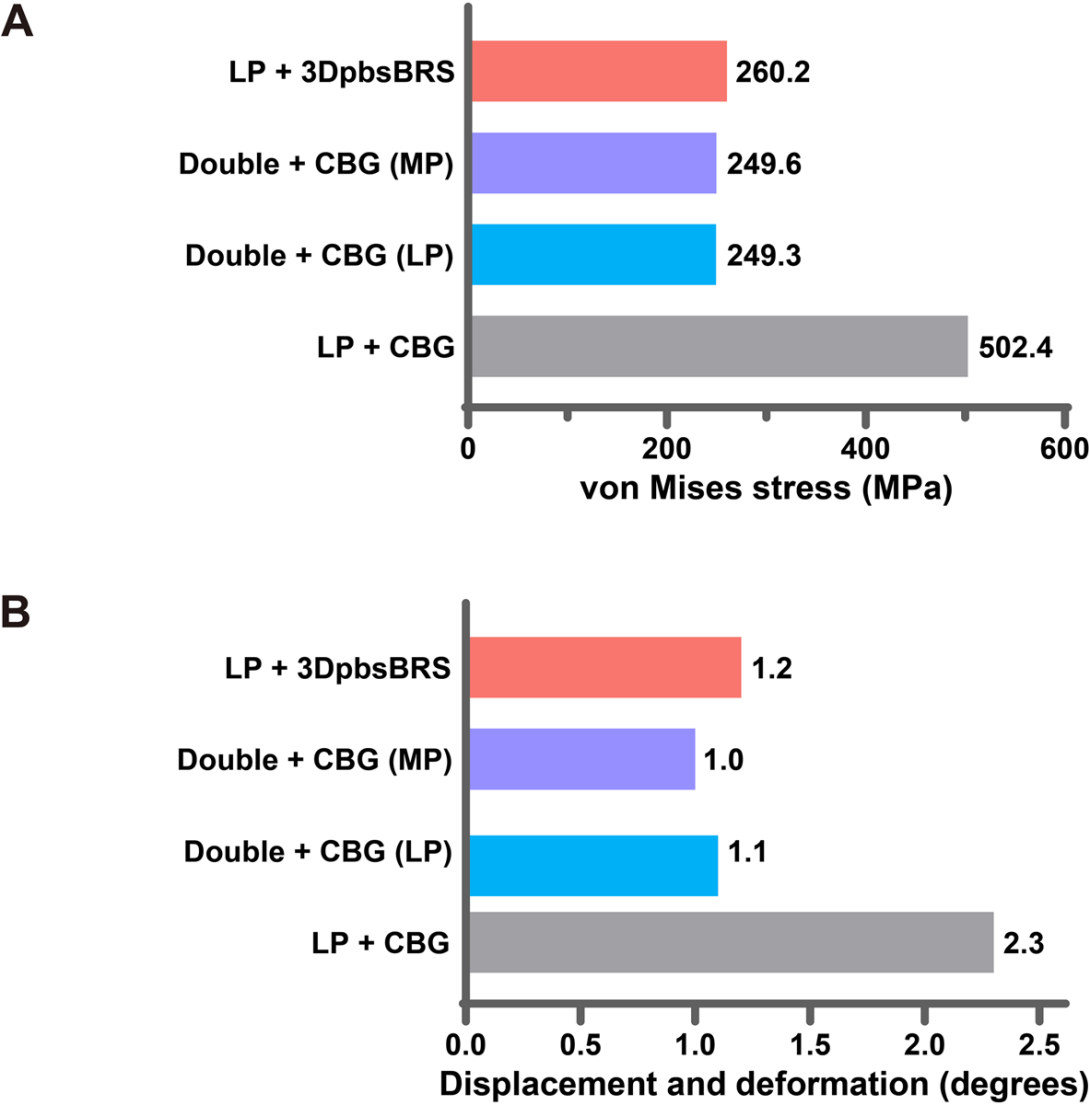
Graphical demonstration of the peak VMS (**a**) and displacement (**b**) in three fixation constructs under 850 N of axial force. VMS, von Mises stress; 3DpbsBRS, 3D-printed, personalized, biomechanics-specific β-TCP bioceramic rod system; LP, lateral plate; MP, medial plate; CBG, cancellous bone grafting; DP, double-plate

We calculated the bending angle of the plate based on the yield strength to evaluate the strength of fixation under axial loading. In the DP + CBG and LP + 3DpbsBRS groups, the bending angles were 1.1° and 1.2°, respectively, which were significantly smaller than the bending angle of 2.3° in the LP + CBG group (Figs. 6 and 7b).

## Discussion

Based on a previous study [15] and our results of finite element analysis (Fig. 7), for the treatment of long bone non-union, it is important to solve the issue of bone reconstruction at the site of non-union and compressive stability of the medial femur. The current study is the first known finite element analysis of LP + 3DpbsBRS to explore the possibility of finding an alternative treatment method that can provide similar medial mechanical support as the MP without causing additional soft tissue and vascular damage.

Multiple studies [16, 29] have reported that double-locking compression plates, an advanced management strategy for femoral shaft non-union, have recognized therapeutic effects. Double-locking compression plates provide constant non-union end compression and the opportunity to remove fibrous scar tissue; thus, they are considered effective for treating femoral shaft non-union with bone defects [30]. Furthermore, regarding mechanical stability, double-locking compression plates are considered to be the best existing method for providing medial mechanical support to the femur because they provide 3D fixation [31, 32]. However, the addition of an MP to the medial femur requires reduced blood supply of the fractured bone ends. Also, the MP itself will cause compression of the periosteum, which will continuously affect the blood supply of the periosteum [31]. Thus, the addition of an MP carries the risk of damaging the blood supply of the bone and inhibiting bone regeneration.

Finite element analysis was used to verify our conjecture that the 3DpbsBRS could provide the same medial mechanical support as the MP and that the combined utilization of LP + 3DpbsBRS could provide the same mechanical support as DP fixation, with less soft tissue damage and blood supply disruption. As shown in Fig. 7, the stress on the LP in the LP + CBG group was 2 times higher than that on the LP in the DP + CBG group, and the bending angle of the LP in the LP + CBG group was also twice that of the LP in the DP + CBG group. In the DP model, we found that some of the stress was dispersed by the MP, resulting in a decrease in the bending angle and stress of the LP. Under axial loading, the LP + 3DpbsBRS and DP groups showed similar results in terms of the bending angle and stress distribution of the steel plate. This series of results indicates that the combined application of LP + 3DpbsBRS provides stability, creating an excellent mechanical environment with limited micromotion for non-union repair and, thus, promoting indirect healing of the non-union [33]. Moreover, the 3DpbsBRS in the LP + 3DpbsBRS group dispersed the medial stress during the treatment of long bone non-union, resulting in less stress on the LP and providing greater shear resistance. The entire plate fixation system showed more stability than LP + CBG and stability equivalent to that of the DP + CBG model. Furthermore, in the LP + 3DpbsBRS group, exposing the non-union end allows removal of fibrous scar tissue and filling with the 3DpbsBRS, when entering from the original incision.

An ideal bone graft substitute should provide a 3D structure to support bone cells, stem cells, and bone ingrowth during degradation and treatment. To avoid these problems, β-TCP bioceramics have been widely used in bone regeneration grafts, which have been proposed for the treatment of bone defects and tested in clinical and animal models [29–31]. The structure of porous bioceramics promotes the growth of fibrovascular tissue, followed by bone apposition on the porous inner surface. Meanwhile, porous bioceramics have also exhibited superior biocompatibility, osteoconductivity and resorption characteristics and are associated with a low infection risk [32]. Degrading β-TCP could also release large amounts of sulphate (SO_4_^2−^) and calcium (Ca^2+^) ions, which are key inorganic salts for forming new bone. β-TCP bioceramics are more biodegradable than hydroxyapatite and can be completely replaced by new bone tissues [32]. In terms of mechanical properties, porous bioceramics tend to perform poorly. β-TCP bioceramics activate cells and signals for the development of new bone and degradation of the implanted material and support the pressure side of the bone. Dense bioceramics have an elastic modulus ranging from 180 MPa to 1.0 GPa and exhibit excellent mechanical properties, with a compressive strength of 10–80 MPa [34]. Recent studies [34–36] have revealed that the compressive strength of β-TCP bioceramics, after 4 weeks of biodegradation, was 24–43 MPa, which is more than 11 times that of porous bioceramics (2 MPa). Moreover, the hardened bone and scar bone tissue at the non-union ends could be cleared and the β-TCP bioceramic could guide the necessary nutrients and stem cells to both ends of the non-union for repair. Dense bioceramics provide immediate structural continuity at the non-union site and early postoperative mechanical support at the site of the non-union while protecting the structure of the porous bioceramic so that the porous bioceramic can continue to induce bone formation. Osteogenesis and biodegradation occur simultaneously, and new bone formation is associated with increased mechanical properties until permanent biomechanical support is achieved. Importantly, considering that personalized and precision medicine should always be the most effective treatment for individual patients [37], personalized treatment strategies, such as the 3DpbsBRS, may be a clinical solution for patients with femoral shaft non-union with bone defects. In addition, the 3DpbsBRS can be combined with bioactive molecules, stem cells and exosomesin future research to potentially yield better regenerative functional and therapeutic results [38–40].

Furthermore, this study offers a novel solution; for other types of bone defects at various fracture sites, finite element analysis based on a mechanical model can help produce a personalized and precise 3DpbsBRS. In these multitudinous scenarios, the 3DpbsBRS can not only serve as a biological substitute for bone but also provide 3D support to reduce the use of additional plates, enhance the therapeutic effect and relieve the financial burden of patients.

Of course, our research has its limitations because it is a study based on finite element simulation with some reasonable simplifications. We are now pushing forward with relevant animal experiments as our follow-up research, and we hope to present more evidence to prove that our new method has a good prospect in the future.

## Conclusion

The 3DpbsBRS in the LP + 3DpbsBRS group could replace the MP in the DP + CBG group by providing similar medial mechanical support. Furthermore, avoiding the use of an MP provides better protection of the soft tissue and vasculature. The 3DpbsBRS is expected to provide a personalized clinical solution for individual patients in various situations, based on the idea that the combination of predictive biomechanical computation and 3D printing technology could provide personalized mechanical support, reduced plate use, and better protection of tissue and blood vessels.

## Data Availability

Please contact author for data requests.

## Abbreviations

DP: Double-plate fixation
LP: Lateral plate
MP: Support medial plate
β-TCP: Beta-tricalcium phosphate
3DpbsBRS: 3D-printed, personalized, biomechanics-specific β-TCP bioceramic rod system
VMS: Von Mises stress
CBG: Cancellous bone grafting
CT: Computed tomography
